# Evaluation of the Efficacy of Standardized Uptake Value (SUV)-shape Scheme for Thyroid Volume Determination in Graves’ Disease: A Comparison with Ultrasonography

**DOI:** 10.22038/aojnmb.2016.7401

**Published:** 2017

**Authors:** Yangchun Chen, Si Pei Xie, Fang He, Jianwei Chen

**Affiliations:** 1 Department of Nuclear Medicine, First Hospital of Fujian Medical University, Quanzhou, China; 2 Department of Ultrasound, First Hospital of Fujian Medical University, Quanzhou, China; 3 Department of Endocrinology, First Hospital of Fujian Medical University, Quanzhou, China

**Keywords:** Thyroid volume, Single-photon emission computed tomography, Software, Ultrasonography

## Abstract

**Objective(s):**

In this study, we aimed to evaluate the efficacy of thyroid volume measurement using ^99m^Tc pertechnetate single-photon emission computed tomography (SPECT) images, acquired by the standardized uptake value (SUV)-shape scheme designed by our expert team.

**Methods:**

A total of 18 consecutive patients with Graves’ disease (GD) were subjected to both ultrasonographic and ^99m^Tc pertechnetate SPECT examinations of thyroid within a five-day interval. The volume of thyroid lobes and isthmus was measured by ultrasonography (US) according to the ellipsoid volume equation. The total thyroid volume, determined as the sum of the volume of both lobes and isthmus, was recorded as TV-US (i.e., thyroid volume measured by US) and set as the reference. The thyroid volume was defined according to our SUV-shape scheme and was recorded as TV-SS (i.e., thyroid volume determined by the SUV-shape scheme). The data were analyzed using the Bland-Altman plot, linear regression analysis, Spearman’s rank correlation, and paired t-test, if necessary.

**Results:**

The values of TV-SS (40.2±29.4 mL) and TV-US (43.0±34.7 mL) were not significantly different (t=0.813; P=0.43). The linear regression equation of the two values was determined as TV-US= 1.072 × TV-SS − 0.29 (r=0.906; P<0.01).

**Conclusion:**

The new scheme, i.e., SUV-shape scheme, exhibited potential for the measurement of thyroid volume in patients with GD.

## Introduction

Iodine-131 (^131^I) therapy is an effective, economic, and safe treatment option for patients with hyperthyroidism (1, 2). Several factors can influence the activity of ^131^I upon administration, including its effective half-life, 24-h radioiodine uptake value, and thyroid mass (3). Since the density of a thyroid tissue is 1 g/mL, thyroid volume is equal to thyroid mass.

Several imaging modalities have been used for the measurement of thyroid volume in patients with Graves’ disease (GD), including planar scintigraphy (4), single-photon emission computed tomography (SPECT) (5), positron emission tomography (PET) (6), X-ray computed tomography (CT), ultrasonography (US), and magnetic resonance imaging (MRI) (7).

Thyroid volume is usually determined by ^99m^Tc pertechnetate planar scintigraphy at nuclear medicine departments of China. The error in thyroid volume, measured by planar scintigraphy, determined according to the threshold value, has been reported to range from −30.36% to 39.45% (8).

In a previous study by Van Isselt et al., a threshold level of 45% of the maximum average pixel value was used to draw the outline of the thyroid gland. The mean relative difference between the thyroid volume measured by SPECT and the reference volume was 2.3±30.5% (5). Therefore, thyroid volume determined with respect to the relative threshold value in planar scintigraphy or SPECT is not convenient for use in the ^131^I dosage scheme.

The standardized uptake value-shape (SUV-shape) scheme was designed by our expert team, using a numerical approximation method to delineate drug-avid tumors on PET images. This scheme has been shown to provide a precise measurement of tumor volume in cases where the tumor exhibits a homogeneous drug uptake and when the tumor size is three times greater than the full width at half maximum (FWHM) of the PET system (9).

Thyroid is usually enlarged in patients with GD and hyperthyroidism. In many cases, the thyroid size increases three times larger than the FWHM of the SPECT image. Additionally, ^99m^Tc pertechnetate is taken up homogeneously in typical cases of GD with hyperthyroidism. Therefore, the potential of SUV-shape scheme to determine the goiter volume in patients with GD was evaluated in this study.

## Methods

### Patients

A total of 18 consecutive patients with GD and hyperthyroidism (female: 12, male: 6; age: 45.2±14.5 years) were included in this study. The patients underwent both US and ^99m^Tc pertechnetate SPECT imaging within a five-day interval. The serum thyroid-stimulating hormone (TSH) level was evaluated within seven days prior to SPECT/CT imaging. Informed consent forms were obtained from all the patients.

### US measurements

US images were acquired, using a real-time US scanner (iU-Elite, Philips) with an 8.5 MHz linear array transducer. The patients were imaged in the supine position with the neck slightly extended. The thyroid lobes and isthmus were scanned separately. The thyroid volume was computed using the ellipsoid volume formula:

*V*= *πabc*/6 where *a*, *b*, and *c* indicate the maximum length, width, and depth along the three principal axes of thyroid lobes and isthmus, respectively. The thyroid volume was defined as the sum of these volumes and labeled as TV-US (i.e., thyroid volume measured by US).

### SPECT

The patients were administered 185 MBq of ^99m^Tc pertechnetate prior to SPECT image acquisition. Twenty min after the administration of the radiotracer, the SPECT data were acquired in supine position, using a dual-detector gamma camera, equipped with low-energy high-resolution collimators (Infinia Hawkeye 4, GE Healthcare), using the following setting: detector position, opposite direction (180°); energy window center, 140 keV; energy window width, 20%; zoom factor, 1.5; and matrix size, 128×128. A rotation of 360° was achieved with 30 azimuths at 30 s/azimuth.

Following SPECT, the patients were subjected to X-ray CT transmission scan, using the following protocol: energy window, 140 keV; tube voltage, 2.5 mA; and total exposure time, 236 s. The SPECT images were reconstructed by the ordered subset expectation maximization with ten subsets, two iterations, and a Butterworth filter (with a critical frequency of 0.48 cycle/pixel and power of 10.0) with resolution recovery, as well as scatter and CT attenuation corrections. The resolution of the SPECT system, i.e., FWHM, was 8.1 mm, while the voxel size was 2.94×2.94×2.94 mm.

### Measurement of thyroid volume using the SUV-shape scheme

The average voxel count of the parotid gland in the maximal slice of the right parotid gland was defined as the background (Bg) in the corresponding CT images. The border of the thyroid gland was delineated by a threshold (Th). The Bg was designated as the initial Th. The mean voxel count of the border was recorded as Cb, while the mean count inside the border was recorded as Ci.

When Cb ≥ (Ci + Bg)/2, the border was considered as that of thyroid. When Cb < (Ci + Bg)/2, Th was reset as (Ci + Bg)/2, the border was re-delineated, and Cb and Ci of the new border were calculated. This procedure of recalculation was repeated until a Cb value ≥ (Ci + Bg)/2 was achieved.

The thyroid volume, labeled as TV-SS (i.e., thyroid volume determined by the SUV-shape scheme), was defined as the product of the number of voxels inside the border and voxel volume. The mean value, standard deviation, skewness, kurtosis, and coefficient of variation (CV) of count/voxel within the thyroid volume were calculated. Ci/Bg and TV-US/TV-SS ratios were also measured.

### Statistical analysis

Statistical analysis was performed, using linear regression analysis, Spearman’s rank correlation, paired t-test, and Bland-Altman plot, if necessary. First, the normality of data distribution was evaluated. The data which did not follow a normal distribution were presented as median values (interquartile range). P-value ≤ 0.05 (two-tailed) was considered statistically significant.

The relative error (RE) was calculated using the following equation introduced in the literature (10):


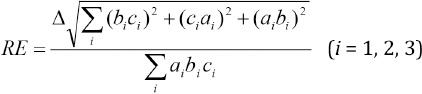


where a1, b1, and c1 represent the maximum length, width, depth of the right lobe, a2, b2, and c2 represent the maximum length, width, and depth of the left lobe, and a3, b3, and c3 represent the maximum length, width, depth of isthmus of thyroid of the right lobe, left lobe, and isthmus of thyroid, respectively. The REs at *Δ*= 1 or 2 voxels (i.e., 2.94 or 5.88 mm) were recorded as *δ* and 2*δ*, respectively. Also, the thyroid volume measurement error (ME) was defined by (TV-US/TV-SS)–1.

## Results

**Figure 1:**
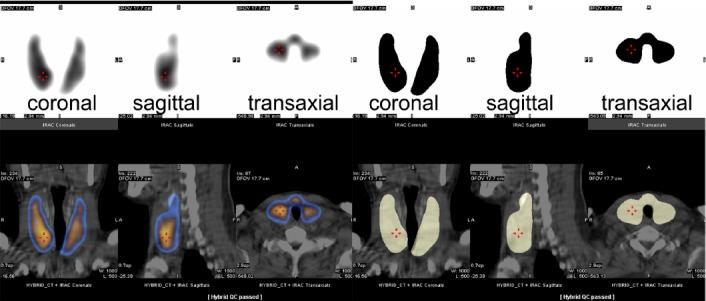
Simultaneously imaged slices of a diffuse enlarged thyroid in a patient with Graves’ disease (GD). Images on the top left show the pertechnetate image acquisition data including those of the coronal, sagittal, and transaxial slices. Images on the top right are the pertechnetate images processed using the SUV-shape scheme. Images at the bottom represent the fusion imaging of images in the top row and the corresponding low-dose CT images. The coronal slice shows the increased length and width of thyroid lobes, with both lobes extending from the superior border of thyroid cartilage to the superior border of clavicle. The sagittal slice shows the increased depth of the right lobe. The transaxial slice shows the enlarged isthmus of thyroid

The thyroid glands exhibited a high and homogeneous uptake of the radiotracer (Figure 1). In contrast, the parotid glands were faintly visualized. The ratio of thyroid/parotid voxel counts, i.e., Ci/Bg, was 19.3±14.0. No uptake activity was observed in the esophagus. The mean TV-US and TV-SS values were 43.0±34.7 and 40.2±29.4 mL, respectively.

**Figure 2:**
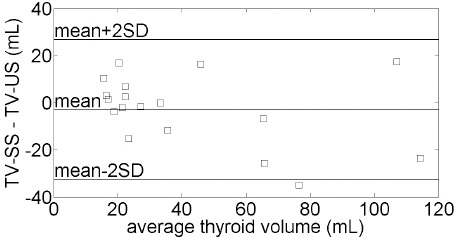
Bland-Altman plot of thyroid volume (TV) in 18 patients with Graves’ disease (GD), measured using SUV-shape (SS) scheme and ultrasonography (US). The standard deviation of the difference between the two measurements (TV-SS – TV-US) is 18.3 mL

As seen in the Bland-Altman plot (Figure 2), the difference in volume measurements between the two methods for most thyroid glands (17/18) was within two standard deviations. However, the volume measurements of only one of the thyroid glands (1/18) exhibited a difference greater than two standard deviations (TV-US vs. TV-SS: 94 vs. 59.1 mL). There was no significant difference between the mean TV-US and TV-SS values (t=0.813; P=0.43).

**Figure 3:**
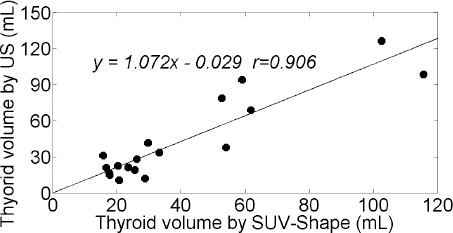
Linear regression plot of thyroid volume in 18 patients with Graves’ disease (GD), measured using the SUV-shape scheme and ultrasonography (β: 1.072±0.125; c: -0.029±6.175; r: 0.906; P<0.01)

The linear regression equation of the relationship between the two measurements was TV-US= 1.072 × TV-SS − 0.29 (r=0.906; P<0.01), as shown in Figure 3. Based on the findings, the ratio of TV-US/TV-SS was 1.09±0.48. The skewness, kurtosis, and CV of the count/voxel within the thyroid volume were −0.287±0.189, −0.643±0.215, and 0.052±0.016, respectively.

**Figure 4:**
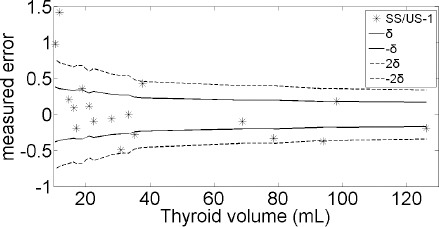
Measurement error (ME) of thyroid volume (TV) determined by the SUV-shape (SS) scheme. The thyroid volume measured by ultrasonography (US) was set as the reference value. The error in measurement was defined by (TV-SS/TV-US)–1

The patients were classified into three groups as follows: group 1, |ME|≤*δ* (9/18 patients, 50%); group 2, *δ*<|ME|≤2δ (6/18 patients, 33%); and group 3, |ME|>2*δ* (3/18 patients, 17%) (Figure 4). The Pearson’s correlation coefficient of the relationship between TV-US/TV-SS and Ci/Bg was −0.154 (P=0.54). Also, the median serum TSH level was 0.019 mIU/L (0.012–0.033 mIU/L). The Spearman’s *ρ* of the relationship between TSH and TV-US/TV-SS ratio was 0.14 (P=0.57).

## Discussion

Thyroid volume is a critical factor influencing the outcome of ^131^I therapy in patients with GD and hyperthyroidism. Accurate measurement of thyroid volume is essential for effective treatment (11, 12). In a previous study, although the volume of multinodularity gland and/or nodular involvement of the isthmus were underestimated by US (in 71 cases), the postsurgical thyroid volume in 21 cases was accurately determined using the applied technique (23%) (13).

In addition, owing to the excellent spatial resolution (< 1 mm), US-based thyroid volume measurement could be used as the reference standard in patients with GD. In this study, we found a major correlation between thyroid volume measurements using the SUV-shape scheme and US measurements (r=0.906). In fact, no significant difference in the mean values was observed between the two methods (P>0.05).

Image noise and/or partial volume effect (PVE) can result in tumor inhomogeneity during imaging (14). In the present study, without the correction of noise and PVE, the skewness and kurtosis of the count/voxel in thyroid volume, determined by the SUV-shape scheme, also showed apparent thyroid heterogeneity.

However, the CV of the count/voxel in the thyroid volume, determined using the SUV-shape scheme, was 0.052±0.016, which is comparable to that obtained in uniform slices by Seret and Forthomme (15). This finding confirms that the activity distributions observed in the thyroid glands in the present study were homogeneous.

The results of thyroid volume measurement delineated by SUV-shape scheme were robust. Neither the thyroid/parotid voxel count ratio nor the blood TSH level was correlated with the results of SUV-shape scheme measurements in this study. Therefore, the operators should draw the ROI only on the right parotid gland to define Bg; this allowed the SUV-shape scheme to measure the thyroid volume by itself.

However, variation in thyroid size affects the ME of the SUV-shape scheme. The ME of SUV-shape scheme in the present study was 0.09±0.48, which is comparable with the previously reported values (5, 8). In fact, the spatial resolution of SPECT images was a critical variable which affected the ME of the SUV-shape scheme.

Voxel is the minimum spatial unit in an image. The voxel size in the present study (2.94 mm^3^) exceeded the spatial resolution of SPECT (8.1 mm). The acceptable MEs of the SUV-shape scheme for the measurement of thyroid volume were *δ* (0.169–0.377) and 2*δ* (0.338–0.754), according to the REs of the corresponding radial lines, which were 2.94 and 5.88 mm, respectively.

In the present study, despite the fact that the MEs of the SUV-shape scheme for 50% (9/18) of the thyroid volume were within the range of ±δ, the application of SUV-shape scheme for personal therapy remains unfeasible. With improvements in the spatial resolution of SPECT (16), the SUV-shape scheme will become feasible for thyroid volume measurements, based on SPECT images including those acquired using ^99m^Tc pertechnetate and ^123^I.

### Study limitations

In the present study, the accuracy of thyroid volume measurements using the SUV-shape scheme in patients with GD was only evaluated with the Infinia Hawkeye 4 system at our department. The accuracy of such measurements should be further evaluated using other SPECT systems by varying the imaging reconstruction parameters.

## Conclusion

In conclusion, upon comparison with ultrasonography, the SUV-shape scheme was found to exhibit potential as a semi-automated method for the SPECT/CT image-based assessment of thyroid volume in patients with GD and hyperthyroidism.
